# Deslorelin Slow-Release Implants Delay Ovulation and Increase Plasma AMH Concentration and Small Antral Follicles in Haflinger Mares

**DOI:** 10.3390/ani11061600

**Published:** 2021-05-28

**Authors:** Martim Kaps, Carolina T. C. Okada, Camille M. Gautier, Jörg Aurich, Christine Aurich

**Affiliations:** 1 Artificial Insemination and Embryo Transfer, Department for Small Animals and Horses, Vetmeduni Vienna, Veterinärplatz 1, 1210 Vienna, Austria; martim.kaps@vetmeduni.ac.at (M.K.); Carolina.Okada@vetmeduni.ac.at (C.T.C.O.); camillemarie.gautier@vetmeduni.ac.at (C.M.G.); 2 Obstetrics, Gynaecology and Andrology, Department for Small Animals and Horses, Vetmeduni Vienna, Veterinärplatz 1, 1210 Vienna, Austria; joerg.aurich@vetmeduni.ac.at

**Keywords:** mare, deslorelin, follicular development, anti-Muellerian hormone, antral follicle count

## Abstract

**Simple Summary:**

In horses, oocyte collection followed by intra-cytoplasmatic sperm injection is increasingly used. The yield of oocytes is a limiting factor and depends on the number of follicles present on the ovary during oocyte collection. Therefore, the aim of this study was to analyze the effect of slow-release implants containing the GnRH analogue deslorelin on the number of follicles and on hormones regulating follicular development. Six mares received a deslorelin implant and six mares served as controls. The interval to the first spontaneous ovulation was prolonged in treated mares. The treatment changed the release pattern of the gonadotrophins LH and FSH. Changes in the number of follicles 10 to 15 mm in diameter were detected in deslorelin-treated mares. These changes were also reflected by increasing plasma anti-Muellerian hormone concentrations, a hormone produced by growing follicles. In conclusion, deslorelin implants induce changes in ovarian follicle subpopulations and could be a promising tool for the preparation of mares for assisted reproductive procedures.

**Abstract:**

There is an increasing interest in the manipulation of ovarian follicular populations in large domestic animals because this could prove beneficial for assisted reproductive techniques such as ovum pick-up (OPU). The aim of the present study was to evaluate the effects of deslorelin slow-release implants (SRI) on the interovulatory interval, antral follicle count (AFC), number of follicles of different size ranges and plasma anti-Muellerian hormone (AMH) concentration in mares. To synchronize their estrous cycles, Haflinger mares (*n* = 12) were treated twice with a PGF_2__α_ analogue. One day after the second injection (day 0), mares received a 9.4 mg deslorelin SRI (group DES, *n* = 6) or 1.25 mg deslorelin in a short-acting formulation (CON; *n* = 6), respectively. Regular transrectal ultrasonography of the genital tract was performed and blood samples were collected for the analysis of progesterone, AMH and gonadotrophins. The interval from implant insertion to the first spontaneous ovulation was 23.8 ± 10.5 days in group DES compared to 17.0 ± 3.9 days in group CON (*p <* 0.05). For the concentrations of LH, FSH and AMH, interactions between time and treatment were detected (*p* < 0.05). The AFC and the mean number of follicles with 5 to 10, 10 to 15 and 15 to 20 mm in diameter changed over time (*p* < 0.05). A time x treatment interaction was demonstrated for follicles of 10 to 15 mm in diameter (*p* < 0.05). The changes in this follicular subpopulation were reflected by increased plasma AMH concentration in group DES. In conclusion, 9.4 mg deslorelin implants show minor effects with regard to estrus suppression in mares, whereas the changes in the subpopulation of small ovarian follicles could be a promising tool for preparation of mares for OPU.

## 1. Introduction

In several species, deslorelin slow-release implants (SRI) are a reliable treatment for transient suppression of sexual behavior and fertility in males and females (e.g., dog [1,2], cat [3,4], cattle [5], pig [6]). Treatment with deslorelin in a slow-release formulation induces an initial stimulation of gonadotrophin release and subsequent downregulation of pituitary GnRH receptors. In response, pulsatile gonadotrophin secretion ceases and sexual function may be suppressed but pronounced species differences have been determined (reviewed by Goericke-Pesch 2017 [7]). Although the sensitivity of horses to down-regulation of pituitary GnRH receptors has been questioned [8], a successful suppression of estrus and estrous behavior in association with a transient inhibition of ovarian activity after treatment of Shetland pony mares with SRI deslorelin implants has recently been demonstrated [9]. The mean interval from SRI injection to the first ovulation was close to 40 days with a 4.7 mg deslorelin SRI and 60 days with a 9.4 mg deslorelin SRI in comparison to 20 days in untreated controls. The efficacy of commercially available deslorelin implants in riding type horses with a double to threefold bodyweight of Shetland ponies still has to be determined. 

Prolonged interovulatory intervals may already occur in mares treated with 2.1 mg deslorelin implants for the induction of ovulation [10,11,12]. This treatment was also associated with reduced plasma FSH concentrations and changes in the ovarian follicular population towards more follicles < 15 mm in diameter [13]. With an increasing interest in transvaginal aspiration of ovarian follicles (OPU) and subsequent intra-cytoplasmatic sperm injection (ICSI) in horses [14], methods for manipulating the ovarian follicular population are required. In horses, recovery rates of oocytes from follicles < 15 mm in diameter are considerably higher than from larger follicles [15]. At present, a reproductive stage of the donor mare promising high oocyte recovery rates is achieved either by repeated OPU at a fixed biweekly schedule [16] or by selecting a favorable ovarian state by repeated ultrasound monitoring of the ovaries [17]. Manipulation of the ovarian follicular population with SRI deslorelin implants might be an interesting alternative for preparing mares for OPU procedures. 

It was therefore the aim of this study to evaluate the effects of a 9.4 mg deslorelin SRI on ovarian activity, follicular development and ovulation in mares of riding type size. We hypothesized that deslorelin treatment results in the transient inhibition of ovulation but also changes the ovarian follicular population with an increase in follicles < 15 mm in diameter. As anti-Muellerian hormone (AMH) in mares originates mainly from early antral follicles corresponding to a diameter between 6 and 20 mm [18], we also investigated if changes in the ovarian follicular population in response to deslorelin SRI treatment would be reflected in changes of plasma AMH concentrations.

## 2. Materials and Methods

### 2.1. Animals

A total of 12 non-lactating Haflinger mares aged between 4 and 16 years (7.4 ± 3.6 years, mean ± SD) with a mean bodyweight of 459 ± 36 kg and a body condition score of 5.1 ± 1.1 [19] were available for the study. They were housed as one group in a large outdoor paddock with multiple sheds available and were fed hay twice-daily. Water was permanently available. The mares’ reproductive health was determined by complete breeding soundness examination before the study.

### 2.2. Experimental Design

The study was approved by the Austrian Federal Ministry for Science and Research (animal experimentation permit numbers BMWFW-68.205/0065-V/3b/2018 and BMBWF-68.205/0113-V/3b/2019) and was conducted in Vienna, Austria (longitude 16.4°, northern latitude 48.3°) starting in April. The experiment followed a protocol recently established in Shetland pony mares [9]. Before the start of the experiment, mares were regularly examined by transrectal ultrasonography to ensure physiological cyclic ovarian activity. All mares displayed at least one spontaneous ovulation before the experiment. Mares received two intramuscular injections of the PGF_2__α_ analogue luprostiol in a luteolytic dose (3.75 mg, Prosolvin, Virbac, Bad Oldesloe, Germany; [20]) 12 days apart, to achieve estrus synchronization. One day after the second PGF_2__α_ injection (day 0), mares were randomly assigned to a treatment (DES, *n* = 6) or control group (CON, *n* = 6). Mares of group DES received a slow-release implant (SRI) containing 9.4 mg of the GnRH analogue deslorelin (Suprelorin, Virbac, Vienna, Austria). Implants were always placed subcutaneously at the left side of the horse’s neck. As treatment with slow-release deslorelin implants is associated with a transient increase in gonadotropin release and might induce ovulation, mares of group CON received a single 1.25 mg intramuscular injection of a short-acting deslorelin acetate formulation (Biorelease Deslorelin, Caledonian, Auckland, New Zealand). Mares of both groups were therefore identical with regard to the initial stimulatory effect but differed with regard to the potential inhibitory effect on pituitary gonadotrophin release. All persons involved in the subsequent examinations were blinded with regard to treatment of the mares.

During the first 10 days of the study, in all mares, the collection of a blood sample and examination of the genital tract, including ultrasound, were performed every second day and from day 10 onwards at five-day intervals. When the mares showed signs of estrus (estrous behavior, pronounced endometrial edema, ovarian follicle > 3 cm in diameter), they were again changed to a two-day interval of sampling and transrectal ultrasonography until ovulation (disappearance of the preovulatory follicle, presence of a corpus luteum). In mares of group DES, examinations ceased when their second spontaneous ovulation after SRI insertion was detected. The examination of group CON mares was continued until the second spontaneous ovulation of all mares in group DES was confirmed. At this time, an additional complete breeding soundness was performed. Blood sampling was continued at five-day intervals in all mares until day 90, and additional blood samples were collected on days 180, 270 and 360. 

### 2.3. Ultrasonography

The presence and degree of endometrial edema was evaluated by ultrasound examination (Mindray M9, Mindray, Shenzhen, China) by the same operator and subjectively scored on a scale of 0 (no edema, diestrus-like structure) to 3 (maximum degree of endometrial edema) as described previously [21]. The presence of corpora lutea and ovarian follicles was recorded and the diameter of the largest follicle was measured. Endocrine activity of a visualized corpus luteum was assumed when progesterone plasma concentration (see Section 2.5) was >1 ng/mL. Multiple short video sequences displaying the whole ovary on both sides were stored for subsequent analyses.

### 2.4. Antral Follicle Count

The antral follicle count (AFC) was assessed on days 0, 10, 25, 50, 90, 180, 270 and 360 of the experiment, as described previously [22]. Briefly, both ovaries were scanned from the ovarian pedicle to the ovarian ligament multiple times and video sequences of 5 s were stored in DICOM format. In case of very large ovaries (e.g., the presence of a preovulatory follicle), the ovary was screened in two separate, parallel planes representing the cranial and caudal part of the ovary, to ensure all follicles were pictured on the videos. The video sequences were analyzed picture by picture with the DICOM JiveX Viewer (Visus Health IT, Bochum, Germany), the sequences were compared and all follicles together with their maximal diameter were noted on an “ovarian map”. In addition to the AFC, the number of follicles was separately evaluated for the follicular size classes 0 to 5, 5 to 10, 10 to 15, 15 to 20, 20 to 25, 25 to 30 and >30 mm in diameter.

### 2.5. Endocrine Analyses

Blood samples were collected by puncture of the jugular vein, alternating on the left and right side, into heparinized tubes (Vacuette, Greiner, Kremsmünster, Austria). The tubes were kept at room temperature and centrifuged within 30 min after collection at 1200× *g* and 5 °C for 10 min. The plasma was immediately frozen and stored at −20 °C until further analysis.

The progesterone concentration in plasma was determined with an enzyme-linked immunosorbent assay (Demeditec Progesterone ELISA kit, Demeditec Diagnostics, Kiel, Germany) as described [9]. Plasma was always diluted 1:100 before analysis. The intra-assay coefficient of variation was 5.4%, the inter-assay coefficient of variation was 10.7% and the minimal detectable concentration was 0.01 ng/mL.

Plasma LH concentrations were measured with a radioimmunoassay validated for equine plasma [23]. Highly purified pituitary-derived equine LH (Roser 2001B; University of California, Davis, CA, USA) was used for standards and iodinated as previously described [24,25]. The primary antibody was a mouse anti-bovine LHb monoclonal antibody (Roser 518B7, University of California, Davis, CA, USA [24]) used at a dilution of 1:1.3 million. The sensitivity of the assay was 0.25 ng/mL and the intra- and inter-assay coefficients of variation were 5.7% and 7.7%, respectively.

Concentrations of FSH were determined using a previously validated RIA [23], with slight modifications as described [9]. The sensitivity of the assay was 0.5 ng/mL and the intra- and inter-assay coefficients of variation were 4.1% and 5.4%, respectively.

Plasma AMH concentrations were determined with an enzyme-linked immunosorbent assay (AL-115, Ansh Labs, Webster, TX, USA) as described previously and validated for equine plasma in our laboratory [22]. The intra- and inter-assay coefficients of variation were 3.2% and 5.1%, respectively, and the minimal detectable concentration was 0.046 ng/mL.

### 2.6. Statistical Analysis

Statistical analysis was performed with the SPSS statistics program (Version 26.0, IBM, Armonk, NY, USA). Data were tested for normal distribution with the Kolmogorov–Smirnov test and were all normally distributed. Differences in the time interval from treatment to first spontaneous ovulation and differences in follicle count of all AFC assessments between groups were compared by *t*-test. Differences between groups in the number of mares ovulating or the presence of corpora lutea, respectively, were compared by Chi-square analysis. Differences in concentrations of LH, FSH and AMH in plasma, ovarian follicle count and follicle subpopulations were analyzed by ANOVA with a general linear model (GLM) for repeated measures with the group as between subject factor and time as within subject factor. Correlations between AMH concentration, AFC and the number of follicles of 5 to 20 mm in diameter were analyzed by Sperman–Rho test. Values are given as mean ± SD. A *p*-value < 0.05 was considered significant. 

## 3. Results

### 3.1. Influence of Deslorelin on Estrous Cycle Characteristics until Day 90

**Figure 1:**
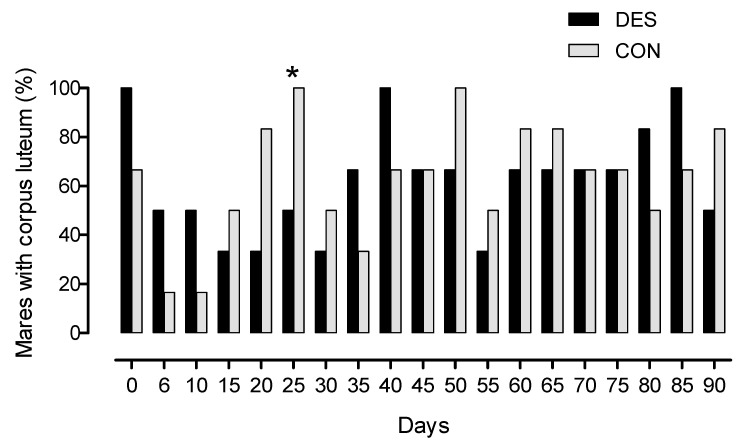
Percentage of mares with corpus luteum (%) in group DES (9.4 mg slow-release deslorelin; black columns, *n* = 6) and group CON (1.25 mg short-acting deslorelin, grey columns, *n* = 6), * indicates significant difference between groups (*p* < 0.05).

**Figure 2:**
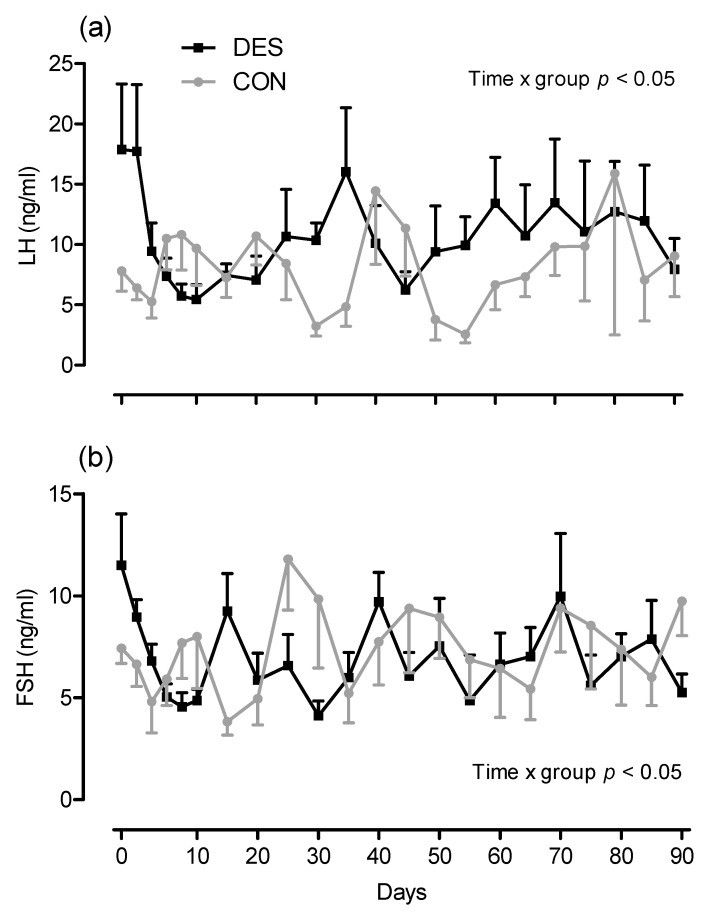
Mean plasma LH (**a**) and FSH (**b**) concentration (ng/mL) in mares of experimental groups DES (9.4 mg slow-release deslorelin; black squares, *n* = 6), and CON (1.25 mg short-acting deslorelin, grey circles, *n* = 6). Information on statistical analysis is given in the figure.

On day 0 of the study, in all mares of group DES and four of six CON mares, a corpus luteum was present, and in all these mares, progesterone concentration in plasma was >1 ng/mL. On day 25, the number of mares with a corpus luteum was lower (*p* < 0.05) in group DES than in group CON (Figure 1). The mean interval from treatment on day 0 to the first spontaneous ovulation was 23.8 ± 10.5 days in group DES compared to 17.0 ± 3.9 days in group CON (*p* < 0.05). Five mares of group DES showed prolonged intervals from treatment to the first spontaneous ovulation (days 34 *n* = 2, 35 *n* = 1, 27 *n* = 1, 24 *n* = 1) compared to group CON mares. In one DES mare, the first ovulation was detected already on day 13 of the study. In the CON mares group, ovulations occurred on days 12 (*n* = 2), 19 (*n* = 2) and 20 (*n* = 2). The concentrations of LH and FSH neither differed over time nor between groups, but a significant time x treatment interaction was shown (*p* < 0.05; Figure 2).

### 3.2. Influence of Deslorelin Treatment on Antral Follicle Count, Follicle Subpopulations and Plasma AMH Concentration until Day 360

**Figure 3:**
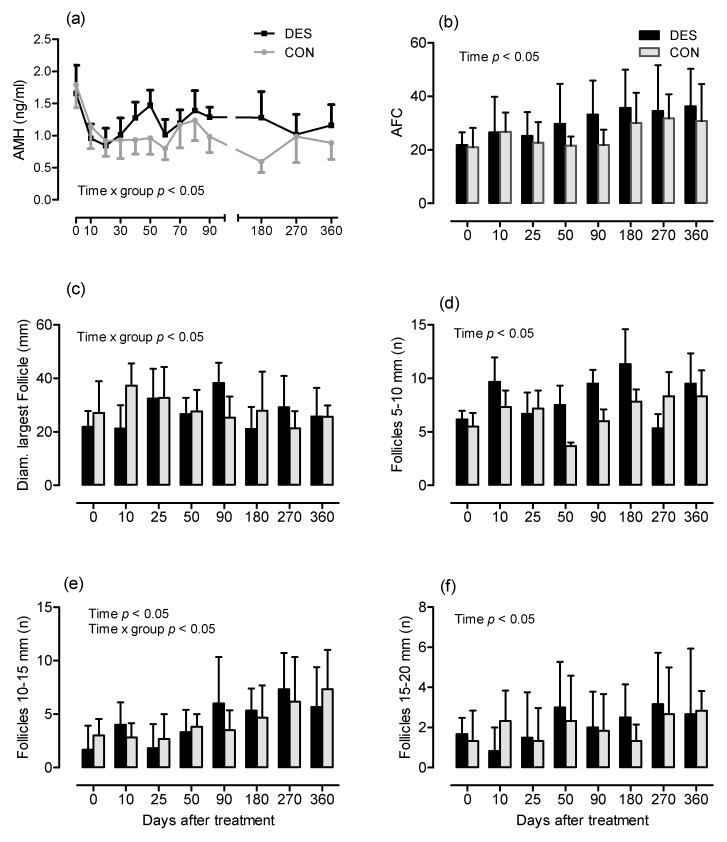
(**a**) Plasma AMH concentration (ng/mL) in mares of experimental groups DES (9.4 mg slow-release deslorelin; black squares, *n* = 6) and CON (1.25 mg short-acting deslorelin, grey dots, *n* = 6). (**b**) Antral follicle count (AFC), (**c**) diameter of the largest follicle and mean number of follicles of different size-ranges, (**d**) 5 to 10 mm, (**e**) 10 to 15 mm, (**f**) 15 to 20 mm in group DES (9.4 mg slow-release deslorelin; black columns, *n* = 6) and CON (1.25 mg short-acting deslorelin, grey columns, *n* = 6).

**Figure 4:**
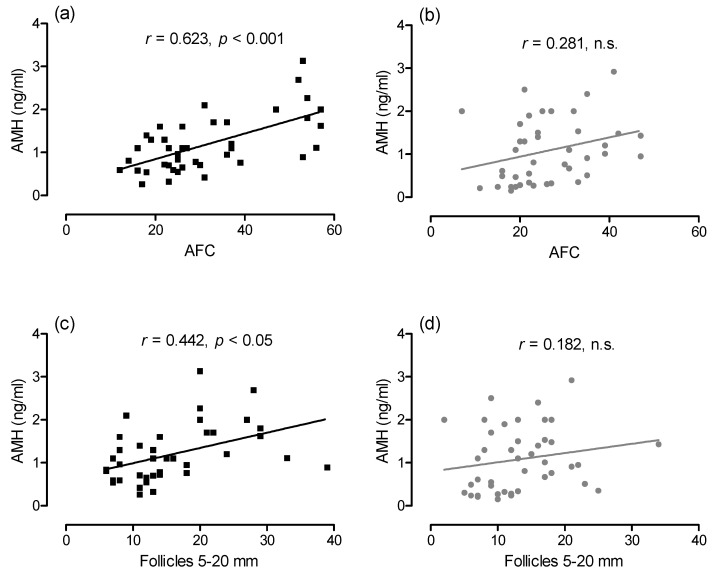
Correlation of AMH and antral follicle count (AFC) in mares of experimental group DES ((**a**); 9.4 mg slow-release deslorelin black squares), and CON ((**b**); 1.25 mg short-acting deslorelin, grey circles). The AFC was positively correlated (*r* = 0.623, *p* < 0.001) in group DES but not in group CON (*r* = 0.281, not significant [n.s.]). Correlation of AMH and number of follicles 5 to 20 mm in diameter in DES ((**c**), *r* = 0.422, *p* < 0.05) and CON ((**d**), *r* = 0.182, n.s.).

The AFC ranged between 7 and 57 follicles, the mean follicle count of all AFC assessments was 31.3 ± 11.9 in group DES and 26.2 ± 5.6 in group CON and did not differ significantly between groups. The AFC and the number of follicles in size classes 5 to 10, 10 to 15 and 15 to 20 mm in diameter changed over time (*p* < 0.05, Figure 3). With regard to the number of follicles with a size of 10 to 15 mm, a time × treatment interaction existed (*p* < 0.05). No differences with regard to time, group or time × group interaction were detected in the other follicle subpopulations (data not shown). The concentration of AMH in plasma did not differ over time or between groups, but an interaction group × time (*p* < 0.05) was detected (Figure 3a). An interaction between time and treatment (*p* < 0.05) was also present for the mean diameter of the largest ovarian follicle (Figure 3e). In group DES, AMH and AFC were correlated (*r* = 0.623, *p* < 0.001), while there was no correlation in group CON (*r* = 0.281, not significant [n.s.]; Figure 4). The plasma AMH concentration and the number of follicles 5 to 20 mm in diameter were correlated in group DES (*r* = 0.442, *p <* 0.01) but not in group CON (*r* = 0.182, n.s.).

## 4. Discussion

To the best of our knowledge, this is the first study describing a long-term effect of deslorelin SRI originally designed for the inhibition of gonadal function in dogs on ovulation, ovarian follicle populations and plasma AMH concentrations in mares of riding-type size. In agreement with previous studies [9,11,12], treatment with deslorelin implants prolonged the interovulatory interval in mares. In the present study, the mean delay of the first spontaneous ovulation was, however, considerably shorter in comparison to a study in Shetland mares where the same 9.4 mg SRI were used [9]. In dogs, a dose-dependent effect of deslorelin SRI on reproductive functions has been described [2,26] and was also suggested in horses [9]. Treatment with 9.4 mg deslorelin SRI inhibited the pituitary response to injection of a GnRH analogue for more than 60 days in Shetland pony stallions [27], but for less than 45 days in Shetland pony mares [9]. In the mares of the present investigation, the pituitary response to a GnRH analogue after treatment with the deslorelin SRI was not determined. Due to the less-pronounced effect on the interovulatory interval in Haflinger than in Shetland pony mares [9], a shorter effective lifespan of the deslorelin SRI than six weeks in larger mares has to be assumed. Due to the short effectiveness and the high costs of the treatment, the utilization of deslorelin SRI for the management of estrus-related behavioral problems in riding-type horses is thus questioned. 

In this study, although the inhibitory effect of deslorelin SRI on ovulatory activity was short-lasting, long-term effects on follicular populations were detected. The most interesting finding was an increase in the group of follicles between 10 and 15 mm in diameter. This is most likely not only a direct result of changes in pituitary FSH secretion in response to deslorelin but also a consequence of the removal of FSH-suppressing factors of follicular origin, with inhibin being the most important [28]. In the present study, a transient increase in gonadotrophin concentrations was followed by a modulated secretion pattern of FSH for the entire experimental period of 360 days. In mares, growth in response to FSH is already stimulated in follicles with a diameter from 2 mm onwards [29], explaining the long-lasting effects of changes in FSH concentrations in the present study. In this context, it has to be noted that in all follicular size classes, changes in numbers over time were detected independent of treatment. It cannot totally be excluded that in the control group, already the treatment with a short acting, injectable deslorelin suitable for ovulation induction in estrous mares influenced follicular growth. This suggestion is in agreement with a previous study where reduced plasma FSH concentrations together with an increase of the subpopulation of follicles < 15 mm in size were detected in mares treated with 2.1 mg deslorelin implants normally used for the induction of ovulation [13]. The injectable deslorelin formulation used in the control mares of the present study, however, has been shown to induce ovulation without causing GnRH receptor downregulation in horses [13,30], long-term influences are thus highly unlikely.

Increased concentrations of LH, FSH and also AMH in the first days after deslorelin treatment in both control and treatment animals are probably not only an effect of deslorelin on pituitary gonadotrophin secretion but are caused by injection of the PGF_2__α_ analogue luprostiol. A pronounced secretory response of the anterior and posterior pituitary to PGF_2α_ analogues has been described previously in horse mares [20,31,32] and cattle [33]. The present study suggests that such effects in mares are not restricted to the pituitary gland but also influence follicular AMH release. 

In this study, deslorelin-induced changes in ovarian follicular populations were reflected in plasma AMH concentrations in treated mares that were higher than in control mares until 270 days after deslorelin SRI implantation. The main source of AMH in mares are growing follicles of 6 to 20 mm in diameter [18,34]. The follicular population where the most pronounced changes due to deslorelin treatment are detectable is well within this range. Changes in AMH concentrations, however, were more pronounced than those in the ovarian follicular population determined by transrectal ultrasound scanning.

A relationship between AFC and AMH concentrations as in young mares [22] could be confirmed in the sexually mature horses of the present study. Interestingly, this relationship was stronger in the deslorelin-treated than in the untreated control mares. This may indicate that deslorelin treatment induces a reset of the ovarian follicular population. Altogether, the effects of deslorelin SRI on the population of small antral follicles may recommend this treatment as a possible approach for the preparation of mares for OPU. In Zebu beef cows, treatment with deslorelin SRI resulted in effects on small follicles similar as in the mares of the present study for up to 180 days and allowed for the aspiration of a higher number of follicles via OPU [35]. In horses, the number of recovered oocytes after the OPU procedure is one of the most important factors for the successful application of ICSI [36]. Follicles similar to the size of the subpopulation increased in the present study promise the best recovery rates [15,37] because they allow for scraping of a greater proportion of the follicular wall, which is a prerequisite for oocyte recovery in horses [36]. Currently, protocols for the manipulation of the ovarian follicular population in horses are lacking. As they are not commercially available at present, treatments with equine FSH or recombinant equine FSH cannot be performed [38]. It may be the aim of future studies to determine if deslorelin SRI treatment might also improve the oocyte yield in aged mares with low AFC [36]. In Zebu cows, the increased number of aspirated follicles after deslorelin SRI treatment was not associated with increased oocyte recovery rates [35]. Detrimental effects of the prolonged absence of luteal tissue and an associated increased LH pulse frequency have been suggested as the underlying cause [39]. Such problems are unlikely in horses because ovulations were suppressed for a much shorter time than effects on follicular populations were detected. A negative impact on oocyte quality is thus unlikely. The optimal interval between deslorelin implantation and OPU procedures to yield maximal oocyte recovery rates in this species still has to be investigated. 

## 5. Conclusions

In conclusion, deslorelin SRI treatment prolonged the interovulatory interval in mares and induced long-term changes in follicular development also reflected in increased AMH concentration in plasma. Due to the short-lasting effect on ovulation, applicability for estrus suppression in this species is low. In contrast, the effects on follicular subpopulations last much longer and make deslorelin implants a promising tool for the pretreatment of mares with the aim to improve the success rate of several assisted reproductive techniques.
